# A Novel Non-Coherent *Q*-Ary TH-PPM Transceiver

**DOI:** 10.3390/s24010105

**Published:** 2023-12-25

**Authors:** Peng Wang, Jie Tian, Duoye Li, Peng Fei, Xianhua Shi

**Affiliations:** 1 Institute of Electronic Engineering, CAEP, Mianyang 621999, China; 18780525385@163.com (P.W.); tianjie@caep.cn (J.T.); liduoye2023@163.com (D.L.); 2 Department of Electronic Engineering, Rocket Force University of Engineering, Xi’an 710025, China; tianlindou@gmail.com

**Keywords:** secure communication, *Q*-ary, time-hopping, pulse position modulation, non-coherent transceiver, bit error rate

## Abstract

Time-hopping pulse position modulation (TH-PPM) stands out as a secure communication due to the pseudo-random characteristics of its time-hopping sequence. However, the conventional TH-PPM transceiver encounters challenges in implementation, particularly in achieving the requisite high precision for synchronization. This paper introduces a novel non-coherent *Q*-ary TH-PPM transceiver, designed to surpass the Bit error rate (BER) performance of conventional TH-PPM transceivers in scenarios under non-ideal synchronization conditions, which also being straightforward to implement. Firstly, we provide an overview of the conventional TH-PPM transceiver. Secondly, the novel TH-PPM transceiver is introduced. In this context, a novel method for generating the TH-PPM signal is proposed for the transmitter, and a parallel matched-filter algorithm, adapted to the new TH-PPM signal, is presented for the receiver. Subsequently, the investigation delves into an in-depth analysis of BER performance, considering both ideal synchronization conditions and non-ideal synchronization conditions, for both the conventional and the new TH-PPM transceiver. Furthermore, the paper proposes a numerical simulation to validate the theoretical findings. The results demonstrate that the new TH-PPM transceiver outperforms the conventional counterpart by showing better BER performance in scenarios with non-ideal synchronization conditions.

## 1. Introduction

Time-hopping pulse position modulation (TH-PPM) operates on the principle of transmitting information through sequences of multiple pulses in the time domain, characterized by a pseudo-random time-hopping sequence. The TH-PPM technology strategically combines the strengths of time-hopping (TH) and pulse position modulation (PPM) techniques for a synergistic enhancement of security performance. Notably, each TH-PPM symbol spans multiple pulses, distributed in accordance with the time-hopping sequence. These inherent characteristic arguments the security of communication, which makes TH-PPM a valuable technology in the domain of secure digital communication.

PPM is a communication technology that conveys information through discrete pulses in the time domain [1] and assigns multiple information bits to each symbol of *Q*-ary PPM. Known for its high energy efficiency, PPM contributes positively to scenarios with power limitations [2,3,4]. On the other hand, TH stands as a spread spectrum technology endowed with multiple-access capability, achieving through orthogonal time-hopping sequences. The noteworthy common feature between TH and PPM lies in their transmission pulses in the time domain, which makes them compatible for integration as TH-PPM. In 1993, R.A. Scholtz proposed TH-PPM for multiple access. A comprehensive analysis of the transmitter waveform and BER performance of the receiver is provided in [5,6,7,8,9]. Subsequently, in 2000, Win and Scholtz employed TH-PPM as an approach for ultra-wideband (UWB) communication. Then the Federal Communications Commission (FCC) in 2002 allocated a substantial frequency band to UWB, rendering it freely available [10,11,12,13,14,15]. This development enabled the integration of UWB into the civilian sector, with TH-PPM emerging as a vital means within this landscape in the subsequent years [16,17,18,19,20,21]. Existing literature often assumes well-established synchronization when analyzing BER performance [22,23]. However, due to the extremely narrow nature of TH-PPM pulses, achieving reliable synchronization proves challenging for conventional TH-PPM receivers [24,25].

Contributions: In this paper, we propose a novel non-coherent *Q*-ary TH-PPM transceiver that exhibits robust BER performance even under non-ideal synchronization conditions. Therefore, it is characterized by a straightforward implementation. The BER performance is analyzed under ideal synchronization conditions and non-ideal synchronization conditions and compared to the BER performance of conventional TH-PPM. Numerical simulations are utilized to validate the analytical framework presented in this study.

The paper is organized as follows. Section 2 introduces the conventional TH-PPM transceiver and the new TH-PPM transceiver. In Section 3, the BER performance is analyzed under ideal synchronization conditions and non-ideal synchronization conditions, both for the conventional TH-PPM and the new TH-PPM. In Section 4, the numerical simulations of BER performance are proposed. In Section 5, we propose a brief discussion comparing the results of known work in the field of UWB. Section 6 is the summary of this paper.

## 2. System Model

In this section, the conventional TH-PPM transceiver is introduced first. Then the new TH-PPM transceiver is proposed. For simplicity, single-user communication is considered in this work.

### 2.1. Conventional TH-PPM Transceiver

**Figure 1:**
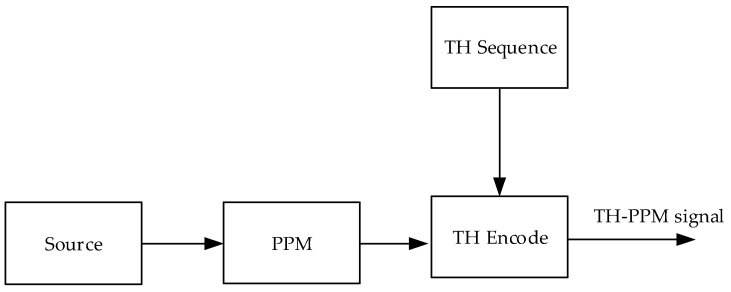
Block diagram of the conventional TH-PPM transmitter.

According to [5], the conventional TH-PPM signal model can be expressed as (1).

(1) $$s^{\left(k\right)}\left(t\right)=\sum_{j}p\left(t-jT_{f}-c_{j}^{\left(k\right)}T_{c}-PPM_{\left[j/N_{s}\right]}^{\left(k\right)}\right)$$

where $s\left(t\right)$ represents the output signal of the TH-PPM transmitter, k represents the user ID, $p\left(t\right)$ represents the pulse waveform of the TH-PPM signal, $T_{f}$ represents the pulse repetition period, $c_{j}$ represents the time-hopping sequence which is limited 1 to $N_{h}$, and $T_{c}$ represents the period of the time-hopping chip, $PPM_{\left[j/N_{s}\right]}^{\left(k\right)}$ indicates the PPM symbol which is determined by the data to be transmitted, and the data to be transmitted changes only every $N_{s}$ hops. The block diagram of the conventional TH-PPM transmitter is shown in Figure 1.

For *Q*-ary TH-PPM, the symbol duration $T_{s}$ is evenly divided into individual time slots $T_{f}$, while each $T_{f}$ is evenly divided into $N_{h}$ time slots $T_{c}$, and then each $T_{c}$ is evenly divided into *Q* time slots ΔT.

**Figure 2:**
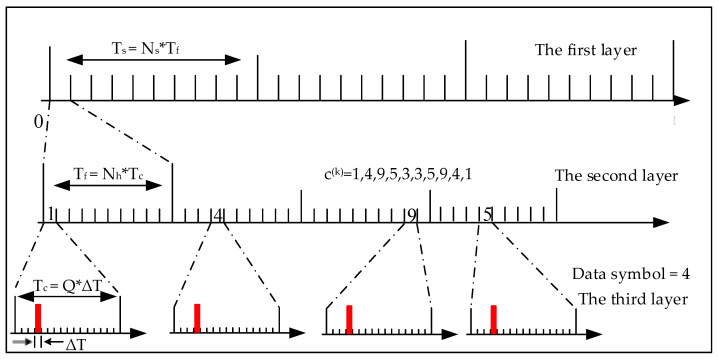
Schematic diagram of 16-ary TH-PPM signal.

Without loss of generality, taking 16-ary TH-PPM as an example, take $N_{s}=N_{h}=10$, take the TH sequence listed as {1,4,9,5,3,3,5,9,4,1}, assuming $PPM_{\left[j/N_{s}\right]}^{\left(k\right)}=4$ is the data to be transmitted, a 16-ary TH-PPM signal is shown in Figure 2.

As shown in Figure 2, the TH-PPM signal can be divided into three layers. The third layer is the same as the conventional *Q*-ary PPM signal structure, with each PPM symbol duration divided into individual time slots, each of which is ΔT, one of the time slots chosen to transmit the PPM pulse modulated by the data symbol. There will be a pulse in the nth ΔT slot when the data symbol which will be transmitted is $n\in \left(1,Q\right)$. For example, when the data symbol will be transmitted is 1, there will be a pulse in the first ΔT slot, and when the data symbol will be transmitted is 2, there will be a pulse in the second ΔT slot, etc. In Figure 2, the data symbol for transmitting is 4, so there is a pulse in the forth ΔT slot.

The middle layer is a layer that embodies the pseudo-randomness of the time-hopping, where $T_{f}$ is the pulse repetition period. Each pulse repetition period $T_{f}$ is divided into $N_{s}$ time-hopping chips. The width of each TH chip is $T_{c}$. In each pulse repetition cycle, the TH sequence pseudo-randomly selects a chip to transmit a PPM symbol. From the perspective of the entire mediate layer, the signal is pseudo-randomly transmitted in the time domain.

The top layer represents the pulse repetition frequency. If the pulse repetition time is $T_{f}$ and the pulse repetition frequency is $N_{s}$, then $T_{s}=N_{s}*T_{f}$ is the duration of a complete TH-PPM symbol. Within each pulse repetition period $T_{f}$, one TH-PPM pulse is transmitted. $N_{s}$ TH-PPM pulses constitute a TH-PPM symbol, and the duration of the TH-PPM symbol is the same as the duration of the *Q*-ary PPM symbol with the same information rate.

For *Q*-ary TH-PPM, we can derive the following relationship from Figure 2.

(2) $$\begin{array}{ccc} \Delta T & = & T_{c}/Q\\ & = & \left(T_{f}/N_{h}\right)/Q\\ & = & \left(\left(T_{s}/N_{s}\right)/N_{h}\right)/Q\\ & = & \frac{T_{s}}{N_{s}\cdot N_{h}\cdot Q} \end{array}$$

**Figure 3:**
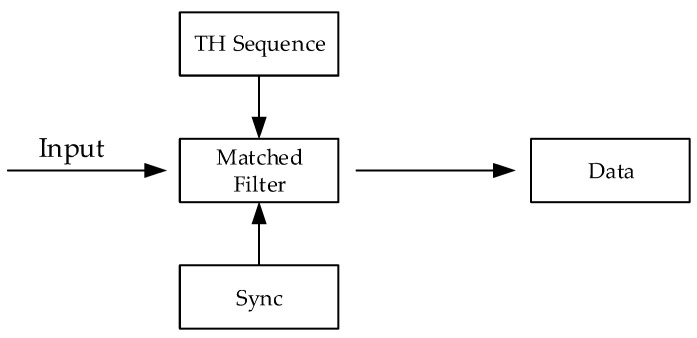
Block diagram for the conventional TH-PPM receiver.

The block diagram of a conventional TH-PPM receiver can be shown in Figure 3.

**Figure 4:**
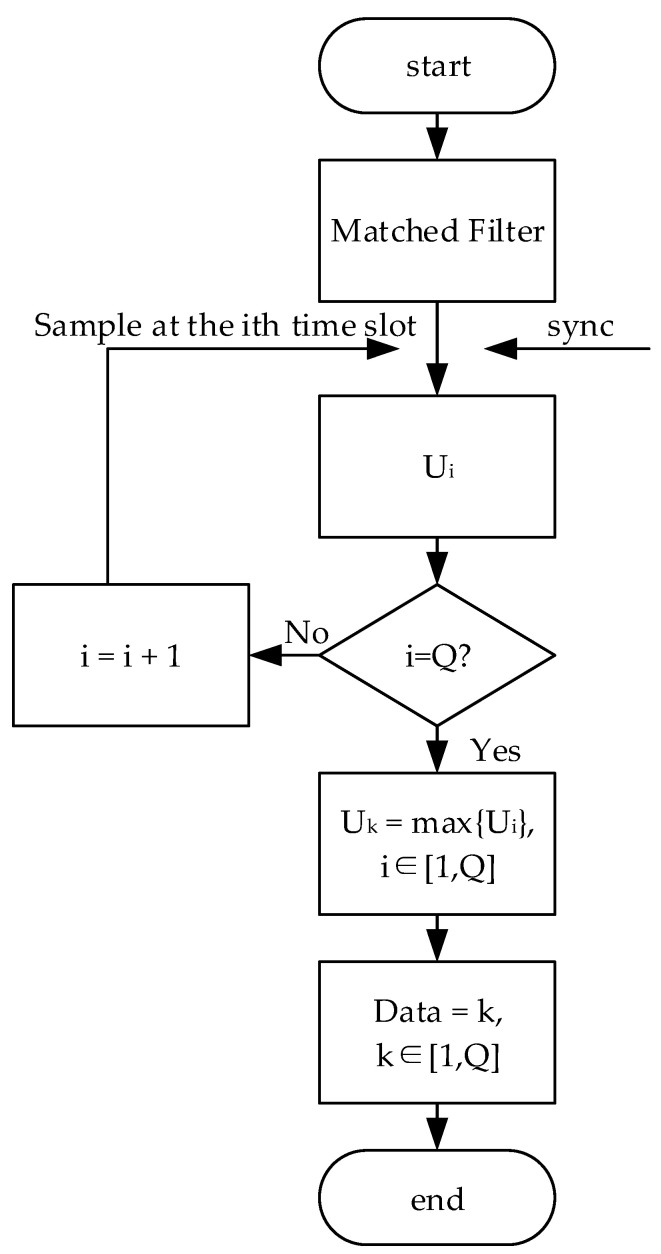
Data decision flow of the conventional TH-PPM receiver.

For conventional TH-PPM receivers, we sample the output of the matched filter at every time slot from 1 to *Q*, which synchronization is required to determine the start of the time slot. Then, if the maximum sampling value is *U_k_*, we can determine the data symbol as *k*, where *k* is limited from 1 to *Q*. The decision flow of the conventional TH-PPM receiver is shown in Figure 4.

1. Start data decision;
2. Sample the values of the matched filter output at time slot 1, 2, …, *Q*;
3. Select the maximum time slot *U_k_* in the sampling values *U*_1_, *U*_2_, …, *U_Q_*;
4. Output data *k* (it is assumed that the maximum sampling value is the *kth* time slot);
5. End decision.

### 2.2. New TH-PPM Transceiver

**Figure 5:**
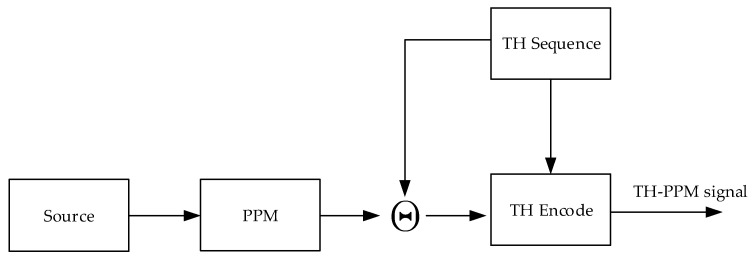
Block diagram of the new TH-PPM transmitter.

The conventional TH-PPM, which is a combination of time-hopping and PPM directly, has the advantage of simplicity and intuition. However, the receiver of conventional TH-PPM requires good synchronization, which is difficult to implement due to the pulse width of the TH-PPM signal being extremely narrow. A minimal synchronization error will lead to significant BER performance degradation. To address this problem, this paper proposed a waveform design method for coupling time-hopping and PPM, which enables certain orthogonality between different TH-PPM symbols, allowing demodulation with parallel matched filter for TH-PPM receiver, which can reduce the time synchronization accuracy requirements and facilitate engineering implementation. The new TH-PPM signal can be represented by Equation (3).

(3) $$s^{\left(k\right)}\left(t\right)=\sum_{j}p\left(t-jT_{f}-c_{j}^{\left(k\right)}T_{c}-c_{j}^{\left(k\right)}\Theta PPK_{\left[j/N_{s}\right]}^{\left(k\right)}\right)$$

where, $s\left(t\right)$ represents the TH-PPM signal. k represents the user ID. $p\left(t\right)$ represents the pulse waveform of the TH-PPM signal. $T_{f}$ represents the pulse repetition period. $c_{j}$ represents the time-hopping sequence, which is limited from 1 to $N_{h}$. And $T_{c}$ represents the width of the time-hopping chip. $PPK_{\left[j/N_{s}\right]}^{\left(k\right)}$ indicates the PPM symbol which is determined by the data to be transmitted, and the data to be transmitted changes only every $N_{s}$ hops. This represents the coupling encoding of the PPM symbol and the time-hopping sequence, which is different from the conventional TH-PPM signal. The block diagram of the new TH-PPM transmitter is shown in Figure 5.

**Figure 6:**
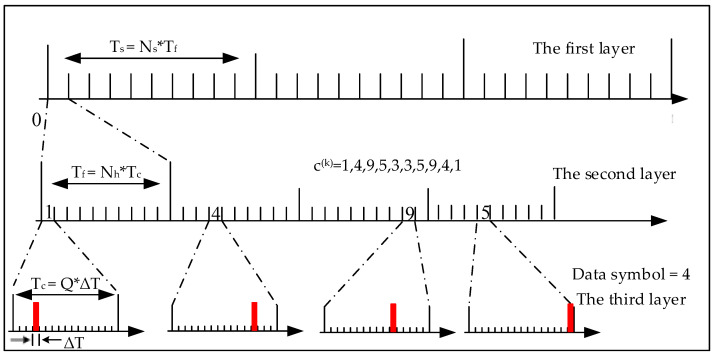
Structure diagram of the new TH-PPM signal.

As same as the conventional TH-PPM signal, the symbol duration $T_{s}$ of the new TH-PPM signal is evenly divided into individual time slots $T_{f}$, while each $T_{f}$ is evenly divided into $N_{h}$ time slots $T_{c}$, and then each $T_{c}$ is evenly divided into *Q* time slots ΔT. The new TH-PPM signal structure is shown in Figure 6.

As shown in Figure 6, compared to the conventional TH-PPM signal in Figure 2, the position of the pulses in the third layer is not only determined by the data to be transmitted but also determined by the time-hopping sequences. In Figure 2, when the data symbol to be transmitted is 4, all the $N_{s}$ pulses in the third layer are located at the fourth ΔT. However, in Figure 6, when the data symbol to be transmitted is 4, the $N_{s}$ pulses in the third layer are located in different ΔT from each other. For example, the first pulse of the new TH-PPM is located in the fourth ΔT of the *Q* slots, while the second pulse is located in the thirteenth ΔT of the *Q* slots. For the new TH-PPM signal, the time-hopping sequence and the PPM modulated symbol are no longer independent. In addition, the new TH-PPM signal doesn’t change the parameters of the pulse width, the number of pulses, and the pulse repetition period. That is, only the position of the pseudo-random occurrence of the signal is changed. Therefore, the time-domain waveform and spectrum of the new TH-PPM signal are the same as those of the conventional TH-PPM signal. For the first layer and the second layer, the new TH-PPM has the same signal structure as the conventional TH-PPM.

**Figure 7:**
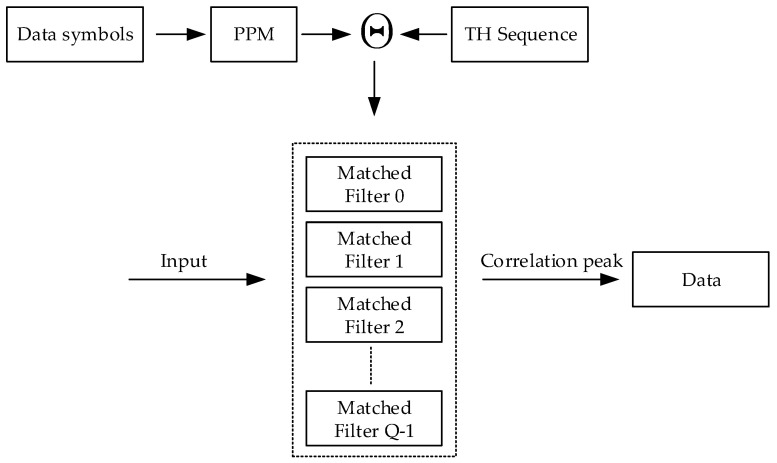
Block diagram of the new TH-PPM receiver.

Due to the orthogonality between different symbols in the new TH-PPM signal, parallel-matched filters can be used for the receiver, as shown in Figure 7. From Figure 7, it can be seen that the new TH-PPM receiver coupled with the time-hopping sequence with the PPM obtains *Q* group-matched filter coefficients, which are used for the *Q*-matched filter. When one of the *Q*-matched filters outputs a correlation peak, we can determine the transmitted data symbol by the corresponding matched filter. In this way, we can reduce the requirement for time synchronization accuracy.

**Figure 8:**
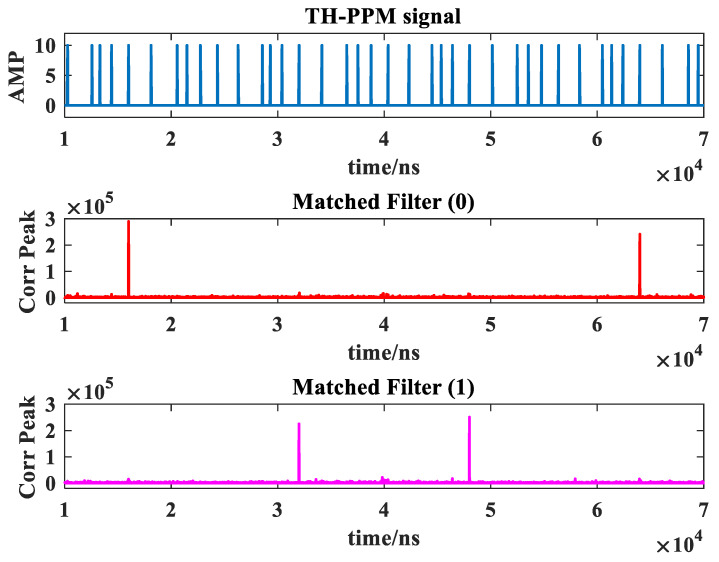
Parallel matched filtering of the new TH-PPM receiver.

Taking 2-ary TH-PPM as an example, the new TH-PPM receiver includes two parallel matched filters. The output of parallel matched filters of the signal is shown in Figure 8.

In Figure 8, there are four data symbols (1, 2, 2, and 1) are transmitted, respectively. When the transmitted data symbol is 1, the matched filter (1) will output a correlation peak, while the matched filter (2) will not output a correlation peak. Therefore, it is possible to determine the received data by judging which matched filter outputs a correlation peak. In this way, we can reduce the requirement for time synchronization accuracy.

**Figure 9:**
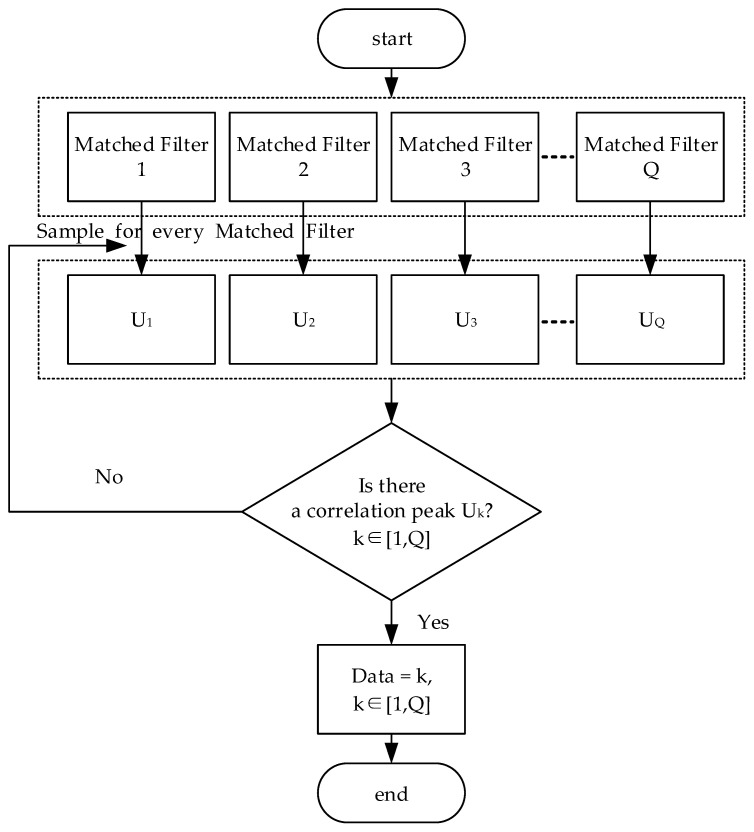
Data decision of the new TH-PPM receiver.

The decision flow of the new TH-PPM receiver is shown in Figure 9.

1. Start data decision;
2. Sample the values of every matched filter from 1, 2, …, *Q*;
3. Determine which matched filter outputs the correlation peak;
4. Output data *k* (it is assumed that the *kth* matched filter is outputting a correlation peak);
5. End decision.

## 3. Performance Analysis

In this section, the BER performance, both of the conventional TH-PPM transceiver and the new TH-PPM transceiver, under ideal synchronization conditions are analyzed first. Then the BER performance under non-ideal synchronization conditions is proposed for constant time offset and jitter, respectively. Furthermore, the BER performance with a change in the shape of the pulse is analyzed.

### 3.1. BER Performance Analysis under Ideal Synchronization Conditions

In this part, it is assumed that the receiver has achieved perfect synchronization, both for the conventional TH-PPM receiver and the new TH-PPM receiver. For the conventional TH-PPM receiver, the TH-PPM pulses are recovered through envelope detection. Then the sampling values are achieved every ΔT slot from the output of the matched filter. The noise envelope sampling without a signal follows the Rayleigh distribution as shown in Equation (4), while the envelope sampling with a signal follows the Rician distribution as shown in Equation (5),

(4) $$p\left(u_{1}\right)=\frac{u_{1}}{\sigma^{2}}\exp\left(-\frac{u_{1}{}^{2}}{2\sigma^{2}}\right)$$

(5) $$p\left(u_{2}\right)=\frac{u_{2}}{\sigma^{2}}\exp\left[-\frac{u_{2}{}^{2}+N_{s}\cdot A_{1}{}^{2}}{2\sigma^{2}}\right]I_{0}\left[\frac{u_{2}\cdot \left(\sqrt{N_{s}}\cdot A_{1}\right)}{2\sigma^{2}}\right]$$

where $u_{1}$ represents the sampling value of the matched filter when there is no TH-PPM pulse in the ΔT slot, $\sigma^{2}$ represents the variance of Gaussian white noise. $u_{2}$ represents the sampling value of the matched filter when there is a TH-PPM pulse in the ΔT slot, $A_{1}$ is the amplitude of the output of the matched filter with a TH-PPM pulse, and $I_{0}\left(\cdot \right)$ is the zero-order Bessel function.

For the conventional TH-PPM receiver, if the sampling value at the time slot without the TH-PPM pulse does not exceed the sampling value at the time slot with the TH-PPM pulse, then the sample is judged to be correct, and the probability is shown in Equation (6).

(6) $$P_{1}=\int_{0}^{u_{2}}p\left(u_{1}\right)du_{1}$$

For a TH-PPM symbol, there are Q−1 time slots without a TH-PPM pulse. Thus, when the Q−1 sampling values do not exceed the sampling value at the time slot with the TH-PPM pulse, the TH-PPM symbol can be determined correctly. The probability is

(7) $$P_{2}=\prod_{i=1}^{Q-1}P_{1}$$

The average of all possible values can be obtained by

(8) $$P_{3}=\int_{0}^{\infty }P_{2}\cdot p\left(u_{2}\right)du_{2}$$

Since the transmitted signal is evenly distributed across the time slots, the probability of obtaining a correct symbol decision is

(9) $$P_{c}=\frac{1}{Q}\sum_{i=1}^{Q}P_{3}=P_{3}$$

Then we can obtain the symbol error rate of the conventional TH-PPM receiver by

(10) $$P_{ew}=1-P_{c}$$

Substituting Equations (4)–(9) into Equation (10) yields

(11) $$P_{ew}=\sum_{k=1}^{Q-1}\left(\begin{array}{c} Q-1\\ k \end{array}\right)\frac{{\left(-1\right)}^{k+1}}{k+1}\exp\left(-\frac{k}{k+1}\frac{N_{s}\cdot A_{1}{}^{2}}{2\sigma^{2}}\right)$$

For $N_{s}\cdot A_{1}{}^{2}/2\sigma^{2}=\log_{2}^{Q}\cdot E_{b}/N_{0}$, we have

(12) $$P_{ew}=\sum_{k=1}^{Q-1}\left(\begin{array}{c} Q-1\\ k \end{array}\right)\frac{{\left(-1\right)}^{k+1}}{k+1}\exp\left(-\frac{k}{k+1}\cdot \log_{2}^{Q}\cdot \frac{E_{b}}{N_{0}}\right)$$

From [20], we have the conversion relationship between the symbol error rate and bit error rate (BER) is

(13) $$P_{eb}=\frac{Q/2}{Q-1}P_{ew}$$

Hence, the BER of the conventional TH-PPM receiver under ideal synchronization conditions is

(14) $$\begin{array}{cc} P_{eb} & =\frac{Q/2}{Q-1}P_{ew}\\ & =\frac{Q/2}{Q-1}\cdot \sum_{k=1}^{Q-1}\left(\begin{array}{c} Q-1\\ k \end{array}\right)\frac{{\left(-1\right)}^{k+1}}{k+1}\exp\left(-\frac{k}{k+1}\cdot \log_{2}^{Q}\cdot \frac{E_{b}}{N_{0}}\right) \end{array}$$

For the new *Q*-ary TH-PPM receiver, there are *Q* parallel matched filters. And every matched filter outputs a sampling value. If the data symbol transmitted is not correlated with one matched filter, the output of the matched filter will be noise, and which sampling value follows the Rayleigh distribution as shown in Equation (4). While the data symbol transmitted is correlated with one matched filter, the output of the matched filter will be a correlation peak, whose sampling value follows the Rician distribution as shown in Equation (5). Also, when the sampling value of the matched filter, which is not correlated with the transmitted data symbol, does not exceed the sampling value of the matched filter correlated with the data symbol, we have (6). If all the sampling values of the Q−1 matched filters, which are not correlated with the data symbol, do not exceed the sampling value of the matched filter correlated with the data symbol, we can obtain a correct symbol decision. Finally, we can have BER of the new TH-PPM receiver as

(15) $$P_{eb}=\frac{Q/2}{Q-1}\cdot \sum_{k=1}^{Q-1}\left(\begin{array}{c} Q-1\\ k \end{array}\right)\frac{{\left(-1\right)}^{k+1}}{k+1}\exp\left(-\frac{k}{k+1}\cdot \log_{2}^{Q}\cdot \frac{E_{b}}{N_{0}}\right)$$

It can be seen that although the demodulation method and the derivation of the BER are different between the conventional TH-PPM receiver and the new TH-PPM receiver, the theoretical formulas of BER are the same under ideal synchronization conditions.

### 3.2. BER Performance Analysis under Non-Ideal Synchronization Conditions with Constant Time Offset

In practical applications, the TH-PPM pulse is very narrow, which needs to achieve high accuracy of synchronization for the receiver. However, it is difficult to implement. Synchronization inaccuracy can be expressed by several factors: constant time offset and jitter. The following are the analyses of the BER performance under non-ideal synchronization conditions with constant time offset for the conventional TH-PPM receiver and the new TH-PPM receiver. Without loss of generality, a time synchronization error factor is defined as μ, representing the ratio of synchronization error to the width of the time slot ΔT. When μ=0, it indicates that the time synchronization is perfect. While μ=0.1, it indicates that there is a slight error in time synchronization. And then μ=1, it indicates that the time synchronization error has exceeded the width of the time slot ΔT.

Due to the data decision flow of the conventional TH-PPM receiver, the BER performance is positively correlated with the sampling values of the output of the matched filter. Therefore, we can obtain the BER performance by analyzing the deterioration of the sampling values output by the matched filter under non-ideal synchronization conditions. Since the accuracy of time synchronization directly affects the envelope of energy within the sampling slot, the envelope of energy within the sampling slot can be expressed as,

(16) $$\left\{\begin{array}{l} A_{1}^{2}{}^{\prime }=\int_{0}^{1-\mu }A_{1}^{2}dt=\left(1-\mu \right)A_{1}^{2},\;0\leq \mu \leq 1\\ A_{1}^{2}{}^{\prime }=0,\;others \end{array}\right.$$

where $A_{1}^{2}{}^{\prime }$ is the actual energy of the envelope within the sampling slot. And $A_{1}^{2}$ representing the energy carried by a TH-PPM symbol. When μ=0, the time synchronization is perfect, and the actual energy of the envelope within the sampling slot is equal to the energy carried by the TH-PPM symbol. When 0≤μ≤1, the SNR of the output of the matched filter can be expressed as,

(17) $$\frac{S^{\prime }}{N^{\prime }}=\frac{N_{s}\left(1-\mu \right)A_{1}^{2}}{2\delta^{2}},\;0\leq \mu \leq 1$$

For $N_{s}\cdot A_{1}{}^{2}/2\sigma^{2}=\log_{2}^{Q}\cdot E_{b}/N_{0}$, there is

(18) $$\frac{S^{\prime }}{N^{\prime }}=\left(1-\mu \right)\cdot \log_{2}^{Q}\frac{E_{b}}{N_{0}},\;0\leq \mu \leq 1$$

Substituting Equation (18) into Equation (14), we have

(19) $$P_{eb}=\frac{Q/2}{Q-1}\cdot \sum_{k=1}^{Q-1}\left(\begin{array}{c} Q-1\\ k \end{array}\right)\frac{{\left(-1\right)}^{k+1}}{k+1}\exp\left(-\frac{k}{k+1}\cdot \left(1-\mu \right)\cdot \log_{2}^{Q}\frac{E_{b}}{N_{0}}\right),\;0\leq \mu \leq 1$$

The analysis is semi-quantitative, for time synchronization errors not only reduce the sampling value by lowering the envelope value of the TH-PPM pulse slot, but also raise the sampling value by raising the envelope value of the noise slot, which further reduces the SNR. Therefore, the analysis is the theoretical lower bound for BER when time synchronization errors exist. The actual BER performance is slightly worse, but it is sufficient for trend analysis.

For the data decision flow of the new TH-PPM receiver, the data symbol is determined by which matched filter outputs a correlation peak. Thus, the start of the time slot is not required. In this way, the SNR of the decision flow will not be reduced by synchronization errors. The demodulation of the new TH-PPM relies on the relative position relationship between multiple pulses within a symbol, while a TH-PPM symbol’s duration is typically about tens of microseconds or less. In such a short time range, it can be assumed that the timing synchronization is fixed and the relative position relationship between the pulses within the symbol remains constant. For parallel matched filters output by the pipeline, the amplitude of the correlation peak will not change. In ideal cases, the BER performance of the new TH-PPM receiver is not affected by the timing synchronization error, and it can still be described by Equation (15).

### 3.3. BER Performance Analysis under Non-Ideal Synchronization Conditions with Jitter

Jitter is another factor that leads to a deterioration in synchronization accuracy. In this part, the BER performance under non-ideal synchronization conditions with jitter is analyzed. We define ε as the factor of jitter by Equation (20),

(20) $$\varepsilon =\frac{\Delta t}{\Delta T}$$

Jitter usually changes randomly over time. For convenience, we define it as the max synchronization error caused by a jitter during a statistical period of time and ΔT is the time slot of the TH-PPM signal model. So, the real jitter changes randomly from 0 to ε in time. When ε=0, it indicates that the jitter is zero and the synchronization is perfect.

For conventional TH-PPM receivers, jitter affects the envelope of energy within the sampling slot, the envelope of energy within the sampling slot can be expressed as,

(21) $$\left\{\begin{array}{l} A_{2}^{2}{}^{\prime }=\int_{0}^{1-\varepsilon }A_{1}^{2}dt=\left(1-\varepsilon \right)A_{1}^{2},\;0\leq \varepsilon \leq 1\\ A_{2}^{2}{}^{\prime }=0,\;others \end{array}\right.$$

where Equation (21) is the same as (16) by replacing μ with ε. According to the analysis from (16) to (19) in Section 3.2, we can have the BER performance with jitter as,

(22) $$P_{eb}=\frac{Q/2}{Q-1}\cdot \sum_{k=1}^{Q-1}\left(\begin{array}{c} Q-1\\ k \end{array}\right)\frac{{\left(-1\right)}^{k+1}}{k+1}\exp\left(-\frac{k}{k+1}\cdot \left(1-\varepsilon \right)\cdot \log_{2}^{Q}\frac{E_{b}}{N_{0}}\right),\;0\leq \varepsilon \leq 1$$

Compare (22) with (19), it is easy to know that, for conventional TH-PPM, the BER performance with jitter is the same as the BER performance with constant time offset when ε=μ. In other words, the BER performance of the conventional TH-PPM is quite sensitive to jitter. The analysis is semi-quantitative, for jitter not only reduces the sampling value by lowering the envelope value of the TH-PPM pulse slot, but also raises the sampling value by raising the envelope value of the noise slot, which further reduces the SNR. Therefore, the actual BER performance is slightly worse with jitter, but it is sufficient for trend analysis.

**Figure 10:**
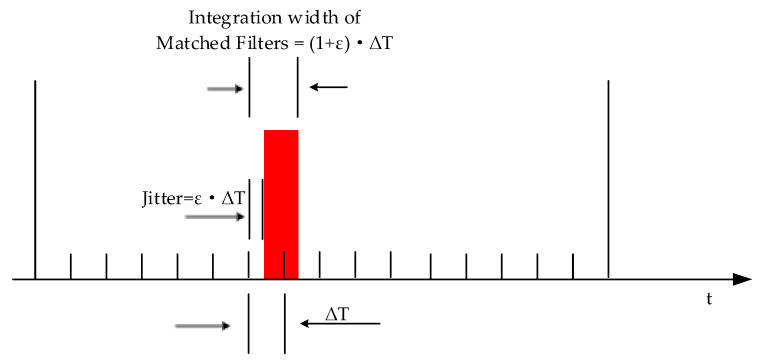
Expand the integration width of matched filters to $\left(1+\varepsilon \right)\cdot \Delta T$.

For the new TH-PPM receiver, thanks to the reduced requirement for synchronization accuracy, we can expand the integration time width of matched filters from ΔT to $\left(1+\varepsilon \right)\cdot \Delta T$, shown in Figure 10. In this way, the matched filter can output a correlation peak without energy loss of the TH-PPM pulse.

Although the matched filter can output a correlation peak without energy loss by expanding the integration width, the noise output of the matched filter will rise to $N^{\prime }=\left(1+\varepsilon \right)\cdot N$. Then the SNR will deteriorate as,

(23) $$\frac{S^{\prime }}{N^{\prime }}=\frac{S}{\left(1+\varepsilon \right)\cdot N}$$

Then, for the new TH-PPM, we can have the BER performance with jitter as (24),

(24) $$P_{eb}=\frac{Q/2}{Q-1}\cdot \sum_{k=1}^{Q-1}\left(\begin{array}{c} Q-1\\ k \end{array}\right)\frac{{\left(-1\right)}^{k+1}}{k+1}\exp\left(-\frac{k}{k+1}\cdot \frac{1}{\left(1+\varepsilon \right)}\cdot \log_{2}^{Q}\cdot \frac{E_{b}}{N_{0}}\right)$$

### 3.4. BER Performance Analysis with Change in the Shape of the Pulse

In pulsed systems, one problem is that the emission of pulsed signals through real band-limited antenna systems leads to a change in the shape of the pulse. It stretches out in time and interference appears between pulses, which leads to synchronization inaccuracies and reduced reception noise immunity. In this part, a brief analysis of the BER performance with a change in the shape of the pulse is proposed. Due to the operation of the time-hopping sequence, the TH-PPM pulses are quite sparse. So, the shape stretches out in time and will not lead to interference between pulses. However, it will reduce the SNR of the receiver. For convenience, we assume that the pulse is stretched out in time uniformly. Without loss of generality, we define δ as the factor of change in the shape of the pulse by (25),

(25) $$\delta =\frac{\tau -\Delta T}{\Delta T}$$

where ΔT is the width of the TH-PPM pulse, and τ is the width of the pulse which is stretched out in time. According to the analysis in Section 3.3, we can have the BER performance of conventional TH-PPM as (26), and the BER performance of new TH-PPM as (27).

(26) $$P_{eb}=\frac{Q/2}{Q-1}\cdot \sum_{k=1}^{Q-1}\left(\begin{array}{c} Q-1\\ k \end{array}\right)\frac{{\left(-1\right)}^{k+1}}{k+1}\exp\left(-\frac{k}{k+1}\cdot \left(1-\delta \right)\cdot \log_{2}^{Q}\frac{E_{b}}{N_{0}}\right),\;0\leq \delta \leq 1$$

(27) $$P_{eb}=\frac{Q/2}{Q-1}\cdot \sum_{k=1}^{Q-1}\left(\begin{array}{c} Q-1\\ k \end{array}\right)\frac{{\left(-1\right)}^{k+1}}{k+1}\exp\left(-\frac{k}{k+1}\cdot \frac{1}{\left(1+\delta \right)}\cdot \log_{2}^{Q}\cdot \frac{E_{b}}{N_{0}}\right)$$

From (22) and (26), it is shown that, for conventional TH-PPM, the BER performance with change in the shape of the pulse is the same as the BER performance with jitter when δ=ε.

From (24) and (27), it is shown that, for new TH-PPM, the BER performance with change in the shape of the pulse is the same as the BER performance with jitter when δ=ε.

The analysis above, which proposes the BER performance of TH-PPM with change in the shape of the pulse, is semi-quantitative, for the assumption that the pulse is stretched out in time uniformly. However, it is sufficient for trend analysis. The weighted receivers can be used to improve the BER performance of TH-PPM with a change in the shape of the pulse. Because there is much literature about weighted receivers [26,27,28], no more expatiation in our work.

## 4. Simulations

In this section, numerical simulations of the BER performance under non-ideal synchronization conditions are proposed, both for constant time offset and jitter.

### 4.1. BER Performance under Non-Ideal Synchronization Conditions with Constant Time Offset

**Figure 11:**
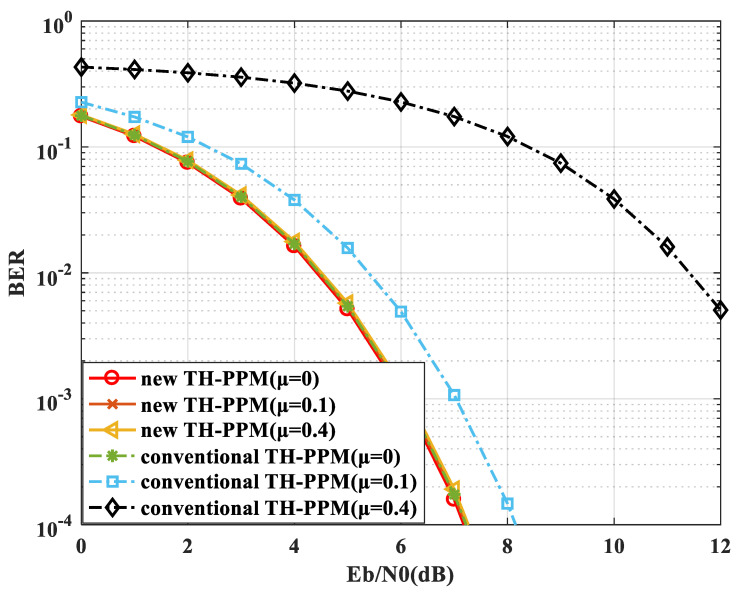
BER performance under non-ideal synchronization conditions with constant time offset.

The following is the numerical simulation of the BER performance under non-ideal synchronization conditions with constant time offset. The simulation parameters are as follows: the *Q* of the *Q*-ary TH-PPM is 16; the pulse repetition period $N_{s}$ is set to 10; and the time-hopping sequence period $N_{h}$ is 10; the time synchronization error factor μ is set to 0, 0.1, 0.4; the $E_{b}/N_{0}$ is set to 0–12 dB; the BER is calculated to $1\times 10^{-4}$; the simulation channel is a Gaussian white noise channel. The simulation result is shown in Figure 11.

A tabular form for performance analysis is proposed in Table 1.

The simulation result shows that as the time synchronization accuracy deteriorates, the BER performance of the conventional TH-PPM rapidly deteriorates. The simulation result is worse than the theoretical analysis mentioned above. The reason is explained in the analysis process. This is because the theoretical analysis belongs to semi-quantitative analysis, which only considers the signal power decrease caused by the decrease, without considering the noise power increase caused by synchronization error. Therefore, the actual SNR deterioration is worse than the analysis mentioned above. However, the trend of the analysis is consistent with the simulation results, which require extreme time synchronization accuracy for the conventional TH-PPM. When the time synchronization error factor is 0.1, the BER performance decreases by about 1 dB, and when the time synchronization error factor is 0.4, the BER performance decreases by about 10 dB. The time synchronization error is larger, the BER performance deteriorates more rapidly. From the simulation, it is shown that the BER performance deterioration does not exceed 1 dB required μ≤0.1, which corresponds to a time synchronization error less than 0.1 of a time slot ΔT. While the ΔT is a nanosecond, even for sub-nanosecond time synchronization is required, which is difficult to achieve.

For the new TH-PPM, the simulation result shows that the time synchronization error has a small impact on the BER performance, which is consistent with the analysis. Compared to the conventional TH-PPM, the new TH-PPM can achieve better BER performance under non-ideal synchronization conditions.

### 4.2. BER Performance under Non-Ideal Synchronization Conditions with Jitter

**Figure 12:**
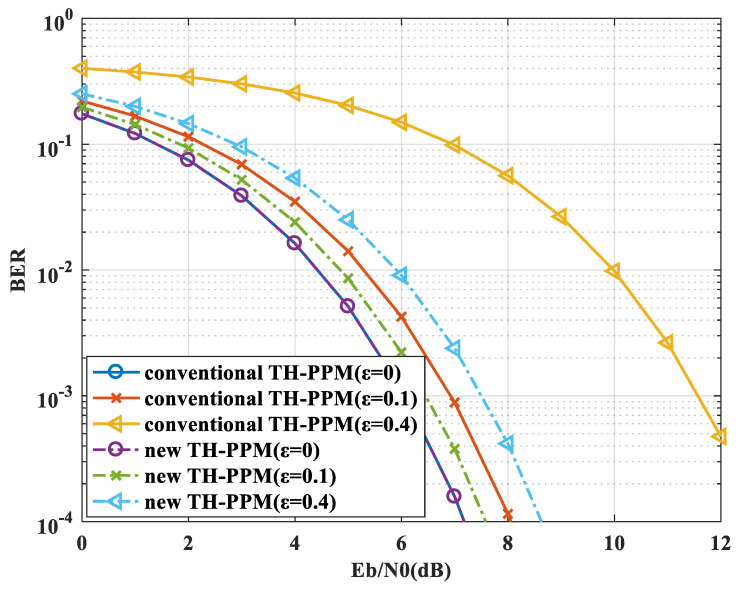
BER performance under non-ideal synchronization conditions with jitter.

The following is the numerical simulation of the BER performance under non-ideal synchronization conditions with jitter. The simulation parameters are as follows: the *Q* of the *Q*-ary TH-PPM is 16; the pulse repetition period $N_{s}$ is set to 10; and the time-hopping sequence period $N_{h}$ is 10; the jitter factor ε is set to 0, 0.1, 0.4; the $E_{b}/N_{0}$ is set to 0–12 dB; the BER is calculated to $1\times 10^{-4}$; the simulation channel is a Gaussian white noise channel. The simulation result is shown in Figure 12.

A tabular form for performance analysis is proposed in Table 2.

The simulation result shows that as the jitter factor increases, the BER performance of the conventional TH-PPM rapidly deteriorates. However, the BER performance of the conventional TH-PPM with jitter is better than that with constant time offset when ε=μ. This is because the real jitter changes randomly from 0 to ε in time, which leads to a better average SNR. Furthermore, the simulation result shows that as the jitter factor increases, the BER performance of the new TH-PPM has also deteriorated. Even so, the BER performance of the new TH-PPM is much better than that of the conventional TH-PPM with jitter, which is consistent with theoretical analysis. According to the analysis in Section 3.4, the BER performance of change in the shape of the pulse is similar to that of the jitter, so we will not propose the numerical simulation with change in the shape of the pulse.

## 5. Discussion

We would like to use the new TH-PPM as a technical for secure communication due to the pseudo-random characteristics of its time-hopping sequence. TH-PPM is well known as a technology for UWB which leads to a variety of literature, a brief discussion compared to the BER performance of known work in the field of UWB is proposed in this section. To the best of our knowledge, there are several ways to improve the BER performance of the UWB.

The first way is to overcome the jitter by designing the waveform of pulse [29,30,31], which was a focus of attention a decade ago. The second way is to improve the accuracy of timing synchronization [32,33,34], which has been a concern in recent years. The third way is about weighted receivers [26,27,28], which can achieve good performance by overcoming the change in the shape of the pulse.

In our work, we proposed a new method different from the known work. We introduce a new structure of the TH-PPM signal model without changing the waveform of the pulse. So, it can be combined with the method of designing a good waveform of pulse to achieve better performance. Furthermore, the receiver in our work, which consists of matched filters, can combine with the method of weighted receivers by changing the filter coefficients with weighted. Then the new TH-PPM can achieve better performance over the change in the shape of the pulse. So, our work is not a replacement for other methods, but a new way which can combine with the other methods to improve the BER performance of TH-PPM.

## 6. Conclusions

In this paper, we propose a new *Q*-ary TH-PPM transceiver, which can achieve better BER performance than the conventional *Q*-ary TH-PPM transceiver under non-ideal synchronization conditions. The signal structure of the new TH-PPM is combined with the time-hopping sequence and PPM, resulting in a certain degree of orthogonally between different TH-PPM symbols. Then, a new TH-PPM receiver composed of *Q* parallel matched filters is proposed. The received signal is simultaneously sent through the *Q* parallel matched filters. And when there is a correlation peak output *kth* matched filter ($k\in \left[1,Q\right]$), the data symbol can be determined to *k*.

The decision flow of the conventional TH-PPM receiver and the new TH-PPM receiver are introduced, respectively. The BER performances are analyzed under ideal synchronization conditions and non-ideal synchronization conditions, both for the conventional TH-PPM receiver and the new TH-PPM receiver. The results show that the new TH-PPM can achieve much better BER performance than the conventional TH-PPM under non-ideal synchronization conditions. Numerical simulations are proposed to verify the analysis of the BER performance. So, the new TH-PPM transceiver can achieve good performance when the synchronization is non-ideal, which is beneficial to implement.

Although the new TH-PPM transceiver can reduce the time synchronization requirements, the complexity of the receiving device will increase significantly with the increase of *Q*, which is equal to the number of Match Filters. When *Q* is small (such as *Q* = 4), it seems that the complexity is acceptable. And we are striving to find ways to reduce the complexity when *Q* is large. Also, the MUI will be considered in future work.

## Figures and Tables

**Table 1:** BER performance under non-ideal synchronization conditions with constant time offset.

*µ*	0	0.1	0.4
**BER deterioration/dB** **(new TH-PPM)**	0	0.1	0.2
**BER deterioration/dB** **(conventional TH-PPM)**	0	1	6

**Table 2:** BER performance under non-ideal synchronization conditions with jitter.

μ	0	0.1	0.4
**BER deterioration/dB** **(new TH-PPM)**	0	0.5	1.5
**BER deterioration/dB** **(conventional TH-PPM)**	0	1	5

## Data Availability

Data are contained within the article.
